# Mutation-associated transcripts reconstruct the prognostic features of oral tongue squamous cell carcinoma

**DOI:** 10.1038/s41368-022-00210-3

**Published:** 2023-01-03

**Authors:** Libo Liang, Yi Li, Binwu Ying, Xinyan Huang, Shenling Liao, Jiajin Yang, Ga Liao

**Affiliations:** 1 General Practice Medical Center, West China Hospital, Sichuan University, Chengdu, China; 2 State Key Laboratory of Oral Diseases, National Clinical Research Center for Oral Diseases, West China Hospital of Stomatology, Sichuan University, Chengdu, China; 3 Department of Laboratory Medicine, West China Hospital, Sichuan University, Chengdu, China; 4 West China School/Hospital of Stomatology, Sichuan University, Chengdu, China; 5 Department of Information Management, Department of Stomatology Informatics, West China Hospital of Stomatology, Sichuan University, Chengdu, China

**Keywords:** Oral cancer detection, Prognostic markers

## Abstract

Tongue squamous cell carcinoma is highly malignant and has a poor prognosis. In this study, we aimed to combine whole-genome sequencing, whole-genome methylation, and whole-transcriptome analyses to understand the molecular mechanisms of tongue squamous cell carcinoma better. Oral tongue squamous cell carcinoma and adjacent normal tissues from five patients with tongue squamous cell carcinoma were included as five paired samples. After multi-omics sequencing, differentially methylated intervals, methylated loop sites, methylated promoters, and transcripts were screened for variation in all paired samples. Correlations were analyzed to determine biological processes in tongue squamous cell carcinoma. We found five mutated methylation promoters that were significantly associated with mRNA and lncRNA expression levels. Functional annotation of these transcripts revealed their involvement in triggering the mitogen-activated protein kinase cascade, which is associated with cancer progression and the development of drug resistance during treatment. The prognostic signature models constructed based on *WDR81* and *HNRNPH1* and combined clinical phenotype–gene prognostic signature models showed high predictive efficacy and can be applied to predict patient prognostic risk in clinical settings. We identified biological processes in tongue squamous cell carcinoma that are initiated by mutations in the methylation promoter and are associated with the expression levels of specific mRNAs and lncRNAs. Collectively, changes in transcript levels affect the prognosis of tongue squamous cell carcinoma patients.

## Introduction

Oral tongue squamous cell carcinoma (OTSCC) is the most common cancer in the oral cavity and is characterized by insidious and high lymphatic metastasis. Consequently, OTSCC has a higher risk and worse prognosis than other oral cancers.^1^ Initially, OTSCC incidence was thought to be higher in the elderly population owing to the accumulation of genetic mutations and risk factors such as long-term smoking, alcohol consumption, and betel nut use.^2–4^ However, recent reports have suggested increasing OTSCC incidence among younger age groups.^5^ The lack of precancerous staging and practical early diagnostic markers for OTSCC has prevented the establishment of an efficient and accurate early warning system. The early warning system, which should be noninvasive or minimally invasive, can be used in high-risk groups to enable a diagnosis before the lesions are fully formed or after surgery. The detection of cancer risk before the lesions are fully formed or before the metastases become established after surgery is of great clinical significance.

Currently, no effective diagnostic technology can meet the needs of early clinical diagnosis. The biggest problem is the lack of molecular diagnostic markers specific to OTSCC. Therefore, it is necessary to identify critical molecular markers with high sensitivity and specificity that can be monitored, screened, and diagnosed in a noninvasive or minimally invasive manner to accurately assess the disease status, improve the prognosis, and provide a better understanding of OTSCC. In addition, the development of new targets is crucial for early diagnosis, precise drug use, accurate prognosis, and understanding of OTSCC pathogenesis. Therefore, searching for practical OTSCC-specific molecular diagnostic markers and establishing rapid, sensitive, simple, and noninvasive diagnostic tests have become the focus of research in OTSCC prevention and treatment.

Using high-throughput sequencing technology, researchers have obtained complete expression profiles, genome-wide data, and genome-wide methylation profiles. Whole-transcriptome sequencing provides access to numerous differentially expressed genes and metabolic pathways; however, genes do not always represent the entire molecular mechanism, and critical signaling pathways are challenging to identify with too many differential genes. Therefore, transcriptome analysis often falls short of the intended research purpose.^6^

DNA methylation is a common alteration at the molecular level and can be readily detected in various states of cell differentiation, especially among cancer cells.^7^ Analyses of DNA methylation have the potential to predict differences in survival and can help detect susceptibility to therapeutic approaches.^8^ In recent years, the use of mRNA markers in serum or tissues as diagnostic or therapeutic targets for OTSCC has been gaining attention because of their effectiveness, utility, and ability to identify mutations with high-throughput screening. Circulating mRNA markers in serum and plasma have been extensively studied as tumor markers.^9^ Long-stranded non-coding RNAs (lncRNAs) play an essential role in the development and prognosis of cancer, but their pathology has been poorly studied.^10^ Transcriptome-wide analysis has shown that 90% of human genetic DNA is transcribed into non-coding RNAs (ncRNAs) lacking protein-coding potential, while lncRNAs are ncRNAs ranging from 200 to 100 kb in length. In malignant tumors, the abnormal expression of numerous lncRNAs is associated with cancer development, including lung, breast, and prostate cancer.^11,12^ Abnormal lncRNA expression has been found in patients with OTSCC and OTSCC metastasis, and lncRNA detection in saliva may potentially be used as a noninvasive rapid diagnostic marker for oral cancer.^12^ DNA methylation plays a vital role in normal mammalian development, but aberrant methylation has been associated with various differentiation-related diseases, including several human cancers. Early epigenetic alterations may contribute to the abnormalities in cellular genes that cause tumorigenesis. Thus, identifying methylation genes may provide a means of preventing and treating OTSCC.^13^

Therefore, in addition to analyzing transcriptomic data from OTSCC and adjacent normal tissue (ANT), this study combined whole-genome sequencing (WGS) and whole-genome bisulfite sequencing (WGBS) to investigate variations in expression profiles. This joint analysis allows greater precision in targeting vital regulatory genes associated with tongue cancer development and progression. We also explored the upstream and downstream regulatory relationships of critical genes. In addition, while some studies have investigated the molecular mechanism of this cancer, few have combined WGS, WGBS, and transcriptome sequencing. The lack of such a joint analysis prompted us to conduct this study. Given the complexity of our analyses and the results obtained, we have created a flow chart for straightforward interpretation (Supplementary Fig. S1).

## Results

### Sample information

Sample information regarding HE-stained tissue sections obtained from five patients is shown in Supplementary Fig. S2. All five samples were squamous cell carcinomas of the floor of the mouth or tongue, and three had lymph node metastasis. The patient clinical information is summarized in Supplementary Table S3.

### Results of the differentially methylated site (DMS) and differentially methylated promoter (DMP) screening

The analysis of different cytosine methylation sites (CG, CHG, CHH, and C) identified 291 mC-, 2 262 mCG-, 1 mCHH-, and 0 mCHG-types among the five groups of samples. The CHH-type DMS was not located in the coding or promoter region, while some C- and CG-type DMSs were. Statistical analysis identified 82 shared C-type DMSs in the coding region and 9 in the promoter region, 1138 shared CG-type DMSs in the coding region, and 160 in the promoter region.

The DMP analysis for different types of cytosine methylation modification sites showed a shared significant difference in methylation sites in all five groups of OTSCC and ANT samples, yielding a total of 5 837 mC-, 1 804 mCG-, 633 mCHG-, and 5 872 mCHH-types (Table 1).

**Table 1:** Number of CG-, CHG-, CHH-, and C-type DMSs in the coding or promoter regions of genes, along with the number of DMPs

Type	CG	CHG	CHH	C
DMS	2 262	0	1	291
DMS located in the coding region of the gene	1 138	0	0	82
DMS in the promoter region	160	0	0	9
DMP	1 804	633	5 872	5 837

### Results of the differential transcriptional analysis

**Fig. 1:**
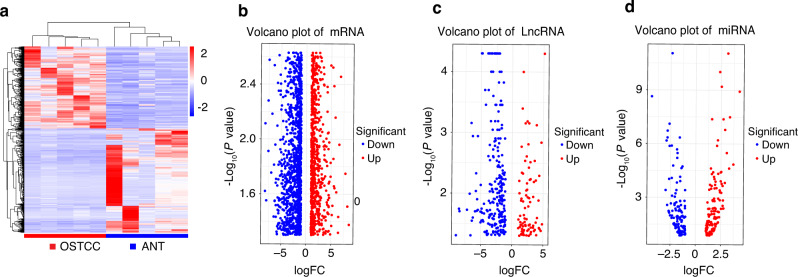
Differential expression of the transcriptome. **a** Heatmap of gene consistency clustering analysis for significant differences between groups of OTSCCs and ANTs. Volcano plots of significantly different expressions of mRNAs (**b**), LncRNAs (**c**), and miRNAs (**d**)

The results of the differential expression analysis of OTSCC and ANT transcripts revealed significant differences (Fig. 1a); 1213 mRNAs were significantly upregulated, and 1 768 mRNAs were significantly downregulated (Fig. 1b). Moreover, 93 lncRNAs were significantly upregulated and 259 lncRNAs were significantly downregulated (Fig. 1c); 128 micro-RNAs (miRNAs) were significantly upregulated, and 117 miRNAs were significantly downregulated (Fig. 1d).

### Methylation promoter and transcriptome correlation

Among the different types of cytosine methylation sites with common DMPs in the five groups of samples, corresponding mRNA transcripts were also significantly different in all the groups; there were 5 CG-type, 7 CHG-type, 40 CHH-type, and 39 C-type DMPs. The results showed that *SDR9C7* and *MAPK8IP2* expression was significantly correlated with the corresponding C-type DMPs; *HAND2* and *SEPP1* expression levels were significantly correlated with the corresponding CG-type DMPs, and *GALNT2* expression was significantly correlated with the corresponding CHH-type DMPs. None of the genes had expression significantly correlated with the corresponding CHG-type DMPs. Based on the human transcription factor target gene data included in the TRRUST database, *HAND2* was identified as a transcription factor with four well-defined target genes (*DBH, GATA4, NPPA*, and *PHOX2A*) and was positively correlated with all of them except *GATA4*.

**Fig. 2:**
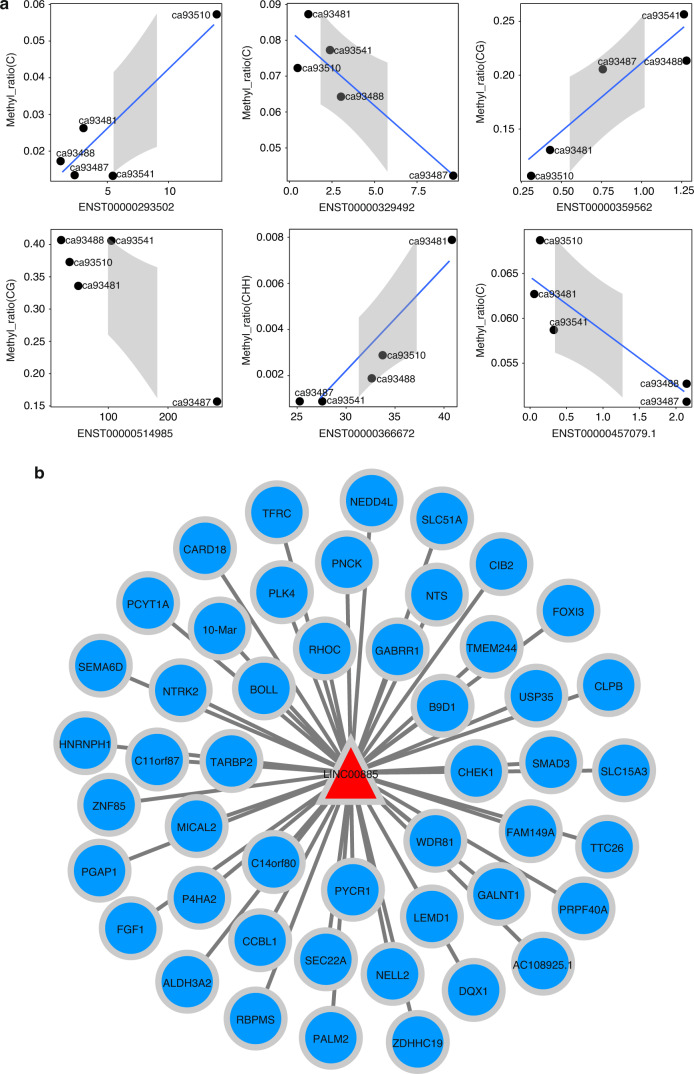
Correlation of methylated promoters with the transcriptome. **a** Correlations of shared DMPs with significantly associated mRNAs and LncRNA. **b** LINC00885 and 45 target genes with a significant association

Among the different types of cytosine methylation sites with common DMPs in the five sample groups, lncRNA transcripts were also significantly different in all groups; there were no CG-type, three CHG-type, eight CHH-type, and six C-type DMPs. The result showed that *LINC00885* expression was significantly correlated with the corresponding C-type DMPs (Fig. 2a). *LINC00885* had 45 target genes, and its expression was significantly correlated with the expression of the 45 target genes (Fig. 2b). The correlations of these six C-type DMPs with gene transcripts and their corresponding promoters are shown in Table 2.

**Table 2:** Six significantly correlated transcripts and their corresponding DMPs

Methylation type	Transcript id	Cor	*P* value	Gene id	Gene name	Gene description
C	ENST00000293502	0.895	0.040	ENSG00000170426	SDR9C7	Short-chain dehydrogenase/reductase family 9C, member 7
C	ENST00000329492	−0.904	0.035	ENSG00000008735	MAPK8IP2	Mitogen-activated protein kinase 8 interacting protein 2
CG	ENST00000359562	0.928	0.023	ENSG00000164107	HAND2	Heart and neural crest derivatives expressed 2
CG	ENST00000514985	−0.899	0.038	ENSG00000250722	SEPP1	Selenoprotein P, plasma, 1
CHH	ENST00000366672	0.933	0.020	ENSG00000143641	GALNT2	Polypeptide N-acetylgalactosaminyltransferase 2
C	ENST00000457079	−0.892	0.042	ENSG00000224652	LINC00885	

### Functional enrichment analysis of shared DMP-related transcripts and differential mRNA

**Fig. 3:**
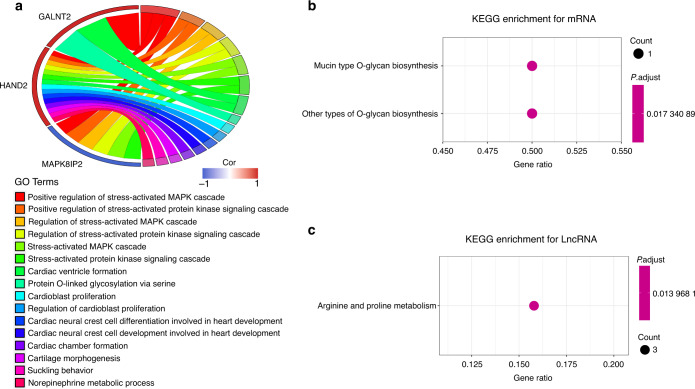
Enrichment analysis of consensus DMP-related transcripts. **a** GO enrichment analysis for mRNA. **b** KEGG enrichment analysis for mRNA, and **c** LncRNA targets

Gene ontology (GO) analysis of mRNA transcripts significantly associated with shared DMPs revealed six pathways containing more than two genes: positive regulation of stress-activated mitogen-activated kinase (MAPK) cascade, positive regulation of stress-activated protein kinase signaling cascade, regulation of stress-activated MAPK cascade, regulation of stress-activated protein kinase signaling cascade, stress-activated MAPK cascade, and stress-activated protein kinase signaling cascade (Fig. 3a). Kyoto Encyclopedia of Genes and Genomes (KEGG) analysis revealed only two signaling pathways (Fig. 3b); the target genes of the lncRNA transcripts correlated with shared DMPs were significantly enriched in arginine and proline metabolism (Fig. 3c).

**Fig. 4:**
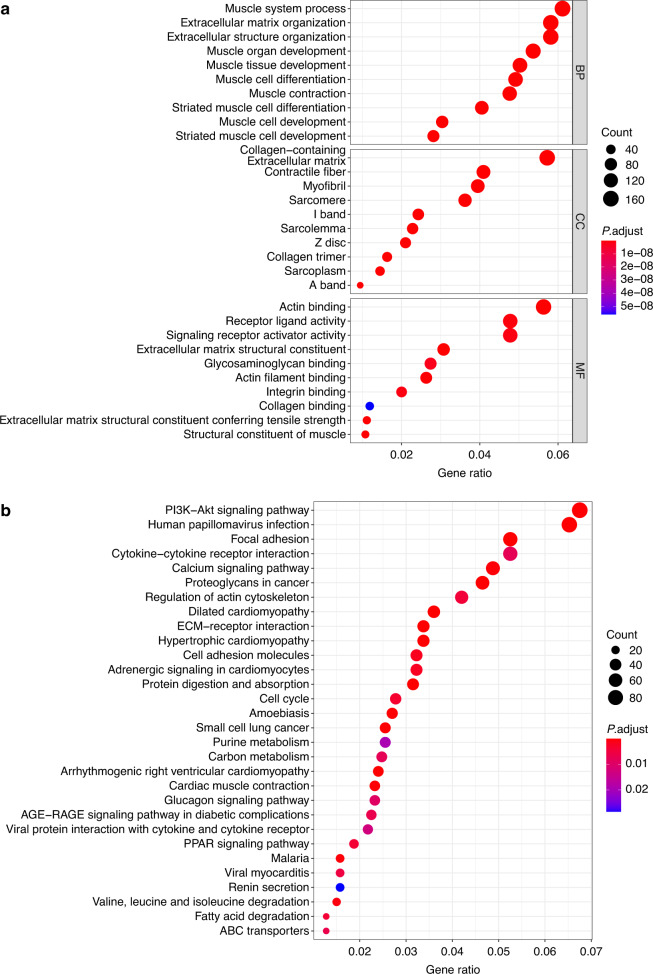
Enrichment analysis of differentially expressed genes. **a** GO enrichment for differentially expressed genes of five paired samples. **b** KEGG enrichment for differentially expressed genes of five paired samples

The results of mRNA transcriptome analysis identified 2981 differentially expressed genes between the five groups of OTSCC and ANT samples. According to GO analysis, the main functions of these genes were the formation of extracellular matrix tissues and structural tissues, the promotion of myoblast development and formation, and the mediation of myofiber movement (Fig. 4a). KEGG analysis revealed the interaction between tumor cells and the extracellular matrix, which constitute the tumor metastasis channel, the formation of adhesion spots, and the formation of proteoglycans in the extracellular matrix of tumor cells (Fig. 4b).

### The Cancer Genome Atlas (TCGA) cohort validation

**Fig. 5:**
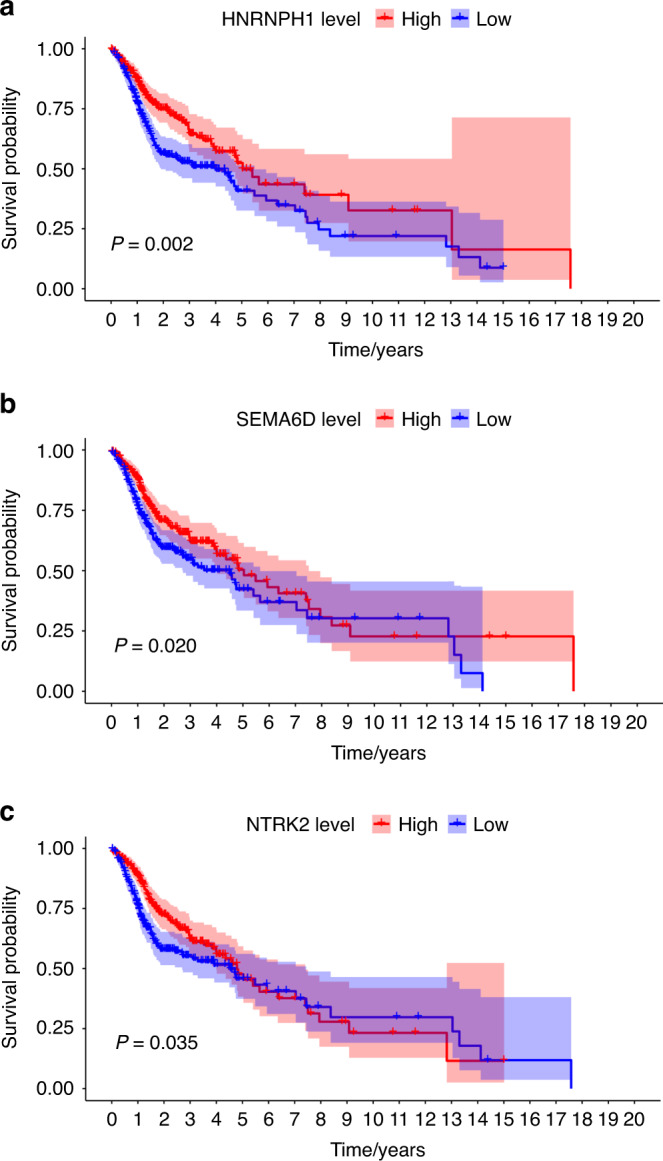
TCGA cohort survival analysis for the Hub genes. **a**–**c** Three target genes (*HNRNPH1*, *SEMA6D*, and *NTRK2*) of LINC00885 were significantly associated with prognosis, as demonstrated by Kaplan–Meier survival curves. Red dots and lines indicate the mRNA high-expression group; blue dots and lines indicate the mRNA low-expression group

Among the *LINC00885* target genes, the expression levels of *HNRNPH1, SEMA6D*, and *NTRK2* were significantly associated with prognosis (Fig. 5a–c). For head and neck squamous cell carcinoma (HNSCC) cohort data from TCGA, we selected eight genes with the most considerable significant differences in the distribution of differentially expressed genes between ANT and OTSCC tissues, as well as in the Kaplan–Meier survival curve results. Among them, *TMPRSS11B* and *GAS2* expression was significantly higher in ANT samples than in OTSCC samples (Supplementary Fig. S4a). Accordingly, patients with high *TMPRSS11B* and *GAS2* expression had a better prognosis than those with low expression. In contrast, *MMP11, TMBIM6, NOMO2, LAMC2, HMGA2*, and *CSF2* expression was significantly lower in ANT samples than in OTSCC samples (Supplementary Fig. S4a). Accordingly, patients with low expression of these genes had a better prognosis than patients with high expression (Supplementary Fig. S4b).

**Fig. 6:**
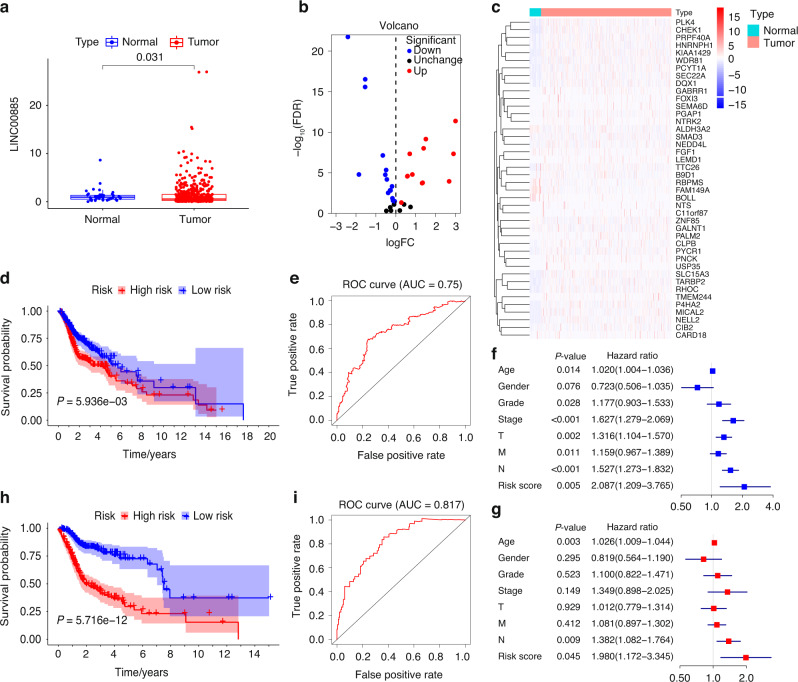
The clinical phenotype–gene model for the survival analysis. **a** Differences in LINC00855 expression in normal tissues compared to that in tumor tissues in the TCGA-HNSCC cohort. **b** Differential expression of LINC00855 target genes in normal versus tumor tissues of the TCGA-HNSCC cohort. Each red dot represents a gene that is upregulated in tumor tissue. Each blue dot represents a downregulated gene. **c** Heatmap showing the expression of each gene in normal and tumor tissues. **d** The Kaplan–Meier survival analysis curve of the prognostic gene signature model predicted the survival of the patients with HNSCC. **e** The ROC curve for the prognostic gene signature model. **f** Forest map based on the results of univariate Cox regression analysis. **g** Forest map based on the multivariate Cox regression analysis results. **h** The Kaplan–Meier survival analysis curve of the clinical phenotype-prognostic gene signature model predicted the survival of the patients with HNSCC. **i** The ROC curve for the phenotype-prognostic gene signature model

**Fig. 7:**
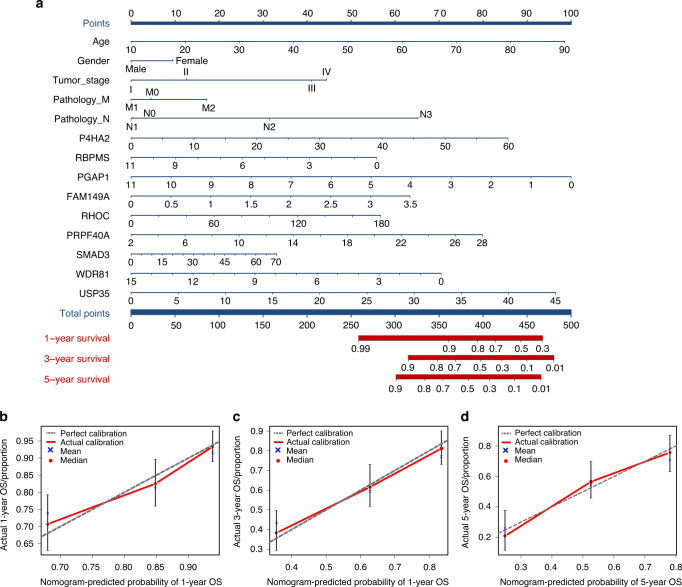
The nomogram contains clinical phenotypes and genes for the predicted prognosis. **a** Each clinical phenotype and gene correspond to the top Points, and the left and right Points are summed to obtain the Total Points. The probability of patient survival at 1, 3, and 5 years can be predicted based on the distribution line of Total Points at the bottom. **b** The nomogram calibration curve for 1-year survival time. **c** The nomogram calibration curve for 3-year survival time. **d** The nomogram calibration curve for 5-year survival time

In the TCGA-HNSCC cohort, the difference in LINC00885 expression between OTSCC and ANT samples was statistically significant (Fig. 6a). Twelve of the LINC00885 target genes were upregulated in cancer tissues, whereas 16 were downregulated (Fig. 6b). The upregulated genes were *PLK4, RHOC, CHEK1, P4HA2, LEMD1, TMEM244, MICAL2, SLC15A3, FOXI3, PNCK*, and *NELL2*. The downregulated genes were *FAM149A, BOLL, RBPMS, ALDH3A2, GALNT1, B9D1, ZNF85 SMAD3, NEDD4L, WDR81, PGAP1, PRPF40A, PALM2, KIAA1429, CLPB, PCYT1A*, and *HNRNPH1* (Fig. 6c). A prognostic signature was developed based on the 45 target genes, using the minor absolute shrinkage and selection operator Cox (LASSO-Cox) analysis shows that the results of the LASSO regression analysis contained two genes (Supplementary Fig. S5a, b). Furthermore, two survival-associated target genes, *WDR81*, and *HNRNPH1*, were selected in the final prognostic signature, and the coefficients were obtained from the LASSO algorithm. The signature calculated the risk score for each patient using the function predict, and each patient was grouped into a high- or low-risk group according to the median risk score. The Kaplan–Meier survival analysis showed that the HNSCC patients in the high-risk group had a significantly shorter OS than HNSCC patients in the low-risk group (Fig. 6d). The area under the receiver operating characteristic (ROC) curve (AUC) of the prognostic signature model (0.75) indicated an acceptable prediction efficiency (Fig. 6e). Single-factor random forest plots showed that patient age, pathological grade, pathological TNM stage, and risk score (RS) reduced survival time and promoted adverse prognostic events (Fig. 6f). Multi-factor random forest plots showed that the model’s RS significantly reduced survival time and promoted adverse prognostic events after association with the patient’s clinical phenotype (Fig. 6g). The combined clinical phenotype-genetic prognostic risk model divided patients into high-risk and low-risk groups based on the median RS. Table 3 shows the risk coefficient corresponding to each clinical phenotype and gene. The LASSO analysis for the clinical phenotype–gene prognostic model shows that there were 14 factors in the regression model (Supplementary Fig. S5c, d). Kaplan–Meier survival curves showed a significant difference in survival time between the high-risk and low-risk groups, with a significantly higher overall 5-year survival rate for patients in the low-risk group than for those in the high-risk group (Fig. 6h). The AUC of the prognostic signature model (0.817) indicated a higher prediction efficiency than the prognostic risk model built on genes alone (Fig. 6i). The final nomogram was constructed based on the factors included in the gene–clinical phenotype-based prognostic characteristics model (Fig. 7a). The closer the red line matches the black diagonal line, the closer the predicted result is to the actual situation (Fig. 7b–d).

**Table 3:** The risk coefficient corresponds to each clinical phenotype and gene

Clinical phenotype	Coefficient	Gene	Coefficient
Age	0.017	P4HA2	0.014
Gender	−0.058	RBPMS	−0.025
Stage	0.189	PGAP1	−0.082
M	0.043	FAM149A	0.073
N	0.287	RHOC	0.001
		PRPF40A	0.028
		SMAD3	0.001
		WDR81	−0.051
		USP35	0.003

### Summary of Cancer Cell Line Encyclopedia (CCLE) lineage analysis

*WDR81* expression in HNSCC tissues was lower than that in many other diseases. In addition, *WDR81* expression was high in skin cancer and myeloma and, conversely, low in diseases such as cervical cancer and teratoma (Supplementary Fig. S6a). *HNRNPH1* expression was significantly lower in HNSCC tissues than in various other diseases but higher in gallbladder cancer and teratoma. Furthermore, *HNRNPH1* was significantly highly expressed in leukemia and embryonal cancer tissues (Supplementary Fig. S6b). Our sequencing results for *WDR81* and *HNRNPH1* are consistent with the mRNA expression interval of the CCLE pan-cancer spectrum results.

The expression levels of *WDR81* and *HNRNPH1* differed significantly in various cell lines of HNSCC. We selected 20 cell lines with the highest and lowest expression for each gene separately for demonstration (Supplementary Fig. S6c, d).

## Discussion

Studies on tongue cancer are lacking; with no apparent marker genes, prognosis and progression are difficult to predict, and low drug sensitivity during treatment is a challenge. Prior research addressing the role of single transcripts in the treatment of OTSCC has failed to elucidate their effects at the genetic level. Reviews examining the relationship between clinical phenotype and tumor have also been unsuccessful at answering this question.^14–17^ The incidence of tongue cancer is increasing^1–4^; however, no credible association between clinical phenotypes and OTSCC prognosis has been found among patients.^18,19^ Therefore, the discovery of novel omics biomarkers is sorely needed, as they may contribute to the prediction of prognosis. This is the focus of this study.

Here, we demonstrated the MAPK cascade involvement of all target genes corresponding to mutant methylation promoters simultaneously present in the tumors of five patients with OTSCC. To date, no other studies have found abnormalities in the expression of genes involved in MAPK cascade signaling pathways in OTSCC tissues. The MAPK pathway mediates cell proliferation, differentiation, and chemotaxis. The negative feedback regulation of the MAPK cascade in cancer cells reduces the sensitivity and efficacy of cancer therapeutic agents.^20,21^ Moreover, the pathway influences essential physiological processes (e.g., neuronal function, immune response, and embryonic development) through regulating gene expression, cytoskeletal protein dynamics, and cell proliferation or apoptosis pathways.^22,23^ Based on our findings and previous reports, we propose a preliminary hypothesis that the MAPK cascade is deeply involved in the biological variation of oral cancer development, migration, and drug resistance. The results of this study suggest that the mutation of methylated promoters triggers aberrant expression of mRNA transcripts, ultimately activating the MAPK cascade. Therefore, if we can target and block specific methylated promoter mutations and the resulting MAPK cascade, we may be able to reduce the likelihood of adverse events in OTSCC.

The occurrence of extracellular matrix heterogeneity is inextricably linked to tumors. The precipitation and mechanical sclerosis of the extracellular matrix are considered key factors leading to tumor infiltration and metastasis.^24–27^ Here, we confirmed that, in tumor samples of patients with OTSCC, an abnormal extracellular matrix is associated with cancer progression. Recent research suggests that activation of the PI3K-Akt signaling pathway promotes epithelial-mesenchymal transition (EMT), ultimately resulting in tumor invasion, metastasis, and drug resistance.^28^ Studies have also confirmed that some specific inhibitors of the PI3K-Akt signaling pathway (e.g., marine drugs) can reverse EMT and thus reduce drug resistance in tumor tissue during treatment.^29,30^ Similar to these previous results, we also noted activation of the PI3K-Akt signaling pathway in OTSCC. Thus, further investigation is needed to determine whether drugs acting on this pathway can inhibit EMT and improve patient prognosis.

Human papillomavirus (HPV)-negative tumors are believed to be associated with the development of oral squamous cell carcinoma, including that of the tongue, and are predictive of poor prognosis and treatment resistance.^31,32^ In our study, HPV infection was the KEGG pathway with the second-highest number of aberrantly expressed transcripts enriched in OTSCC tissue. Another pathway of importance was the calcium signaling pathway, which is involved in crosstalk with reactive oxygen species (ROS) signaling pathways leading to tumorigenesis.^33,34^ Inhibition of calcium signaling can inhibit cancer cell proliferation and metastasis in some cancers.^35^ Consistent with previous findings, our study confirmed the activation of the calcium signaling pathway in OTSCC tissues.

Data from the TCGA-HNSCC cohort were used to verify whether our results aligned with previous extensive sample analyses. Notably, the most differentially expressed transcripts in OTSCC were correlated with prognosis in the TCGA-HNSCC cohort. Nevertheless, the potential heterogeneity between HNSCC and OTSCC, ethnic differences in the patients studied, and differences in sequencing methods can explain why some of our results were inconsistent with the results of the HNSCC cohort analysis. With the use of a larger sample size in future research, the credibility of our findings will also increase.

LINC00885 promotes tumor cell proliferation and invasion.^36^ Current research has focused on breast and cervical cancers, but LINC00885 expression in OTSCC remains unclear.^37,38^ Our findings revealed that LINC00885 is also upregulated in OTSCC. Therefore, the prognostic signature model based on the target gene of LINC00885 has high predictive efficacy in predicting the patient’s prognosis after surgery or treatment. Notably, since both *HNRNPH1* and *WDR81* have risk coefficients less than 0 in the risk profile model, the patients’ risk scores are the absolute value of the actual risk score. In this study, *HNRNPH1* and *WDR81* were highly expressed in OTSCC tumor samples and were protective genes for predicting prognosis. The combined clinical phenotype–gene model has a more reliable predictive efficacy than gene-based models, but more complex information needs to be collected.

Our study found no correlation between mutations occurring in the genome and differences in the transcriptome. However, this does not mean that genes are not mutated in OTSCC tissues or that gene mutations do not affect the transcriptome, thereby leading to functional changes. On the contrary, genomic sequencing of OTSCC tissue samples from five patients showed many genomic mutations in tumor tissues compared to that in ANT samples.

Our study found that 2 mC-, 2 mCG-, and 1 mCHH-type methylation mutations cause aberrant expression of the transcriptome in OTSCC. Mapping such molecular changes to cellular functions revealed differences in MAPK cascade pathways. Further, lncRNAs and their target genes in the variants were used to predict the prognostic risk of patients. Ultimately, such changes lead to cancer development, increased drug resistance, and suboptimal prognosis in patients with OTSCC. Analysis of transcripts showed that five patients with OTSCC had differential genes mainly clustered in pathways with multiple functions. These pathways include deposition and mechanical sclerosis of extracellular matrix tissue, PI3K-Akt signaling pathway leading to EMT, HPV infection, and interaction of calcium signaling with ROS signaling.

In conclusion, this study provides a theoretical basis for follow-up research on experimental etiology or interventions. Targeted blockade of specific methylated promoter mutations and the resulting MAPK cascade may be a new direction for reducing adverse events in OTSCC. The prognostic signature models constructed based on *WDR81* and *HNRNPH1* and the combined clinical phenotype–gene prognostic signature models show high predictive efficacy and can be used to predict patient prognostic risk in the clinical setting.

## Materials and methods

### Sample and data collection

Five patients with OTSCC were enrolled, and paired OTSCC and ANT samples were surgically excised from each patient. After washing off bloodstains with saline while removing non-essential tissues, samples were dried with gauze, cut into tissue blocks less than 0.5-cm thick, and placed into labeled RNase-free cryotubes or EP tubes. The tubes were snap-frozen in liquid nitrogen and stored at −80 °C. Next, we sequenced the whole transcriptome of the ten samples via RNA sequencing (RNA-seq), WGS, and WGBS.

### Differential methylation sites and promoter methylation screening

Based on the WGS results, we screened for mutations co-existing in the five sets of paired samples. We next screened and genetically annotated the differentially methylated regions in all paired samples. We then screened for DMSs and DMPs common to the five sets of paired samples based on the WGBS results. Loci and promoters with statistically significant differences between OTSCC and ANT samples were considered DMSs and DMPs, respectively. Statistical significance was set at *P* < 0.05.

### Differential expression of the transcriptome

We further analyzed and collated whole-transcriptome sequencing data, including lncRNA, mRNA, and miRNA sequences. Differential expression analysis between groups was performed with the OTSCC and ANT samples separately using DESeq2, and software used to detect differentially expressed genes with duplicate samples.^39^ The screening was conditioned on differential ploidy of ≥2 and *P* < 0.05.

### Correlation between DMPs and the transcriptome and gene function prediction

Based on the DMPs shared by the five sets of paired samples, we further analyzed the effect of their modifying effects on the transcripts. Transcript data were obtained through differential genes analysis. We extracted mRNA and lncRNA transcripts with significantly different expressions in the five sets of paired samples during the analysis. Their expression was subjected to Pearson correlation analysis with their corresponding shared DMPs. The relationships between DMPs and transcripts were considered significant at *P* < 0.05, with the absolute value of correlation coefficients greater than 0.9. Screened transcripts were significantly correlated with their corresponding shared DMPs, indicating that DMPs regulated the expression of these transcripts in tumor tissues.

We used the R packages “org.Hs.eg.db”^40^ and “clusterProfiler”^41^ to perform GO and KEGG enrichment analyses of transcripts associated with shared DMPs to determine their functional pathways. Similarly, we performed GO and KEGG enrichment analyses on differentially expressed transcripts co-occurring in the five paired samples to understand how biological functions vary across OTSCC and ANT.

### TCGA cohort validation

We downloaded HNSCC cohort mRNA transcriptome data and follow-up data using the TCGA database. We used Perl software to organize the data initially. We organized the data using the R package “survival”^42,43^ and performed survival analysis with “survminer”.^44^ We verified whether the expression of lncRNA target genes associated with shared DMPs impacts the prognosis of patients with HNSCC. The Least Absolute Shrinkage and Selection Operator (LASSO) can retain the most representative variables, which is valuable for the model’s accuracy.

Consequently, it is considered by researchers an effective high-dimensional predictive regression method to avoid over-fitting of the model variables. First, a Multivariate Cox analysis was performed to determine whether the selected gene is a prognostic factor in patients with HNSCC. We used the LASSO-Cox regression model to show the ideal risk coefficient of each prognostic feature for the genes in the HNSCC prognostic signature model. In addition, the RS was calculated by the function PREDICT. Patients were defined as high risk if their RS was above the median and low risk if their RS was below the median. Risk coefficients were used to distinguish between protective or risk factors and to determine the ability of each factor to affect prognosis. A factor more significant than 0 was considered a risk factor, and a factor less than 0 was a protective factor. A more considerable absolute value indicated that the factor had a more significant impact on prognosis. The next step was to observe whether the prognosis between the two groups was different over time. The Kaplan–Meier survival analysis with a two-sided log-rank test was performed to assess the difference in the prognosis between the two groups. Next, the ROC curve was used to determine the accuracy of the model’s prediction. Next, univariate and multivariate Cox regression analyses were performed to identify the independent prognostic factors for the HNSCC cohort. The survival difference between the high-risk and low-risk groups was stratified based on age, gender, histologic grade, tumor stage, and pathological T/N/M stage. Finally, the prognostic risk of a combined gene–clinical phenotype was modeled similarly.

The prognostic impact of transcripts was verified using box plots showing differential gene expression in cancer and adjacent tissues and Kaplan–Meier survival curves. Ultimately, the nomogram was constructed based on the factors included in the model. Finally, the calibration curve was used to determine how well the predicted results match the formal situation.

### CCLE for showing the pan-cancer and cell lineages

We downloaded gene expression across the cancer spectrum from the CCLE database into the prognostic risk model. We visualized the results to present the expression levels of these genes in each cancer type or different cells of HNSCC.

### Statistical analysis and data processing

For all statistical analyses, statistical significance was set at a *P* < 0.05. All statistical analyses were performed using the software Perl and R. The graphs were plotted based on the packages “ggpubr” and “ggplot2”.^45,46^ Analysis of variance was performed with the program package “edgeR”.^47^ The package “survival” was used for the integration of survival times and ending events,^48^ the package “glmnet” was used for the LASSO regression analysis,^49,50^ and the package “survminer” was used to plot Kaplan–Meier survival curves.^51^ The final patient RS was calculated by the function to predict, whereas the function coef algorithm calculated the coefficients of each factor in the prognostic risk profile model. The plotting of ROC curves and the calculation of AUC values were implemented by the package “survivalROC”.^52^ The plotting of the random forest plot was performed with the package “forestplot,” and the function forestplot was used for plotting.^53^ Finally, the program package “rms” was used to construct the nomogram. Since quantitative numerical data are required in the random forest analysis, we transformed the TNM staging according to the staging values and kept only numerical data. For gender, we defined female as 0 and male as 1. The details of the statistical analysis tools were shown in Supplementary Table S7.

## Supplementary information

supplementary materials
