# Supplementary material for: Mutation-associated transcripts reconstruct the prognostic features of oral tongue squamous cell carcinoma
Source: Int J Oral Sci. 2023 Jan 3;15:1. doi: 10.1038/s41368-022-00210-3 (PMC9807648; doi:10.1038/s41368-022-00210-3)
Supplement: Supplementary file 1 — supplementary materials [file 41368_2022_210_MOESM1_ESM.pdf]

Mutation-associated transcripts reconstruct the prognostic features of oral tongue squamous cell carcinoma

Libo Liang[1], Yi Li[2], Binwu Ying[3], Xinyan Huang[4], Shenling Liao[3], Jiajin Yang[4], Ga Liao[2,5*]

Supplementary materials

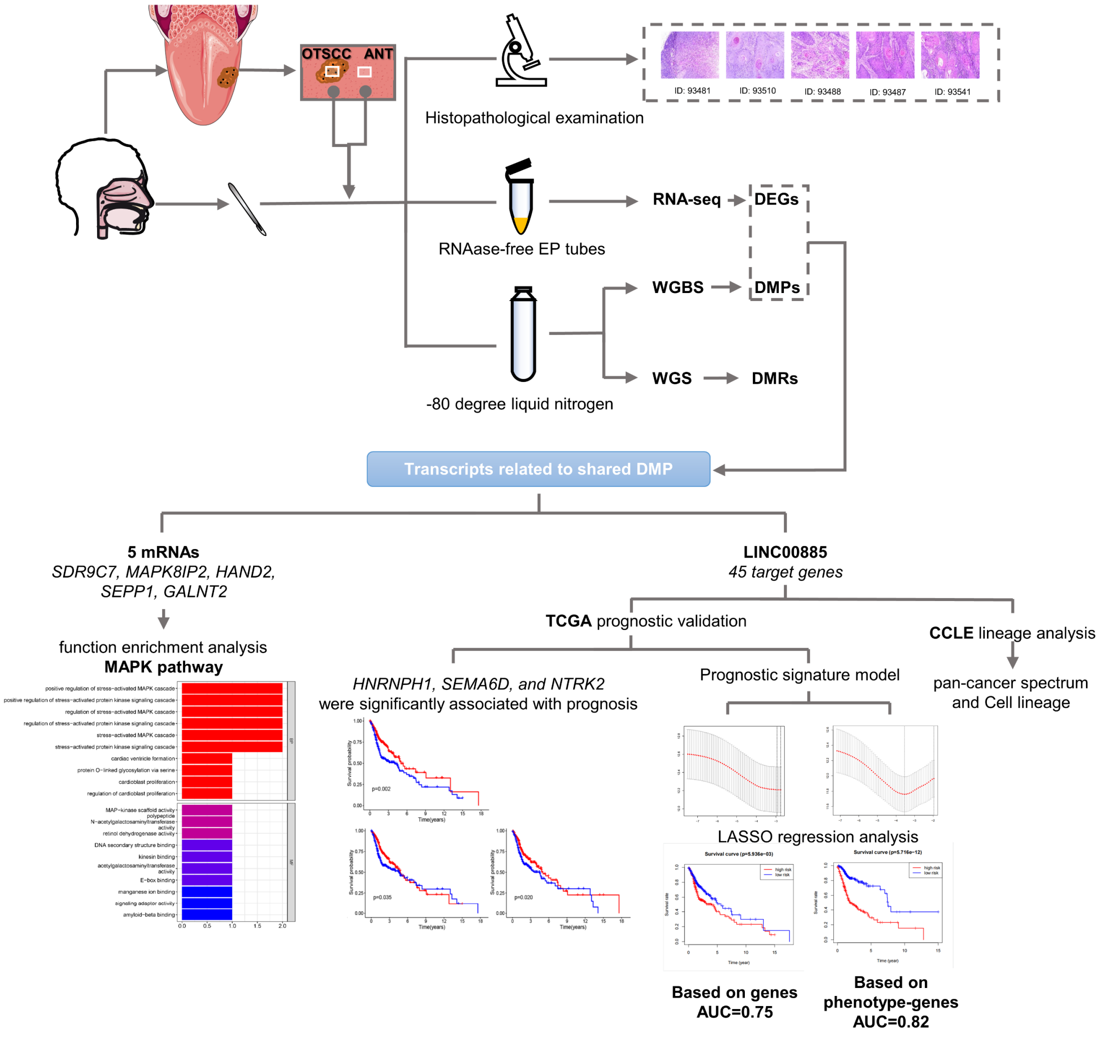

Fig. S1. Flow chart of the experiments and analyses. OTSCCs and ANTs were surgically removed. A total of 10 samples were subjected to RNA-seq, WGS, and WGBS analysis to screen for biomarkers related to prognosis. In addition, the Cancer Genome Atlas (TCGA) and Cancer Cell Line Encyclopedia (CCLE) were used for external validation.

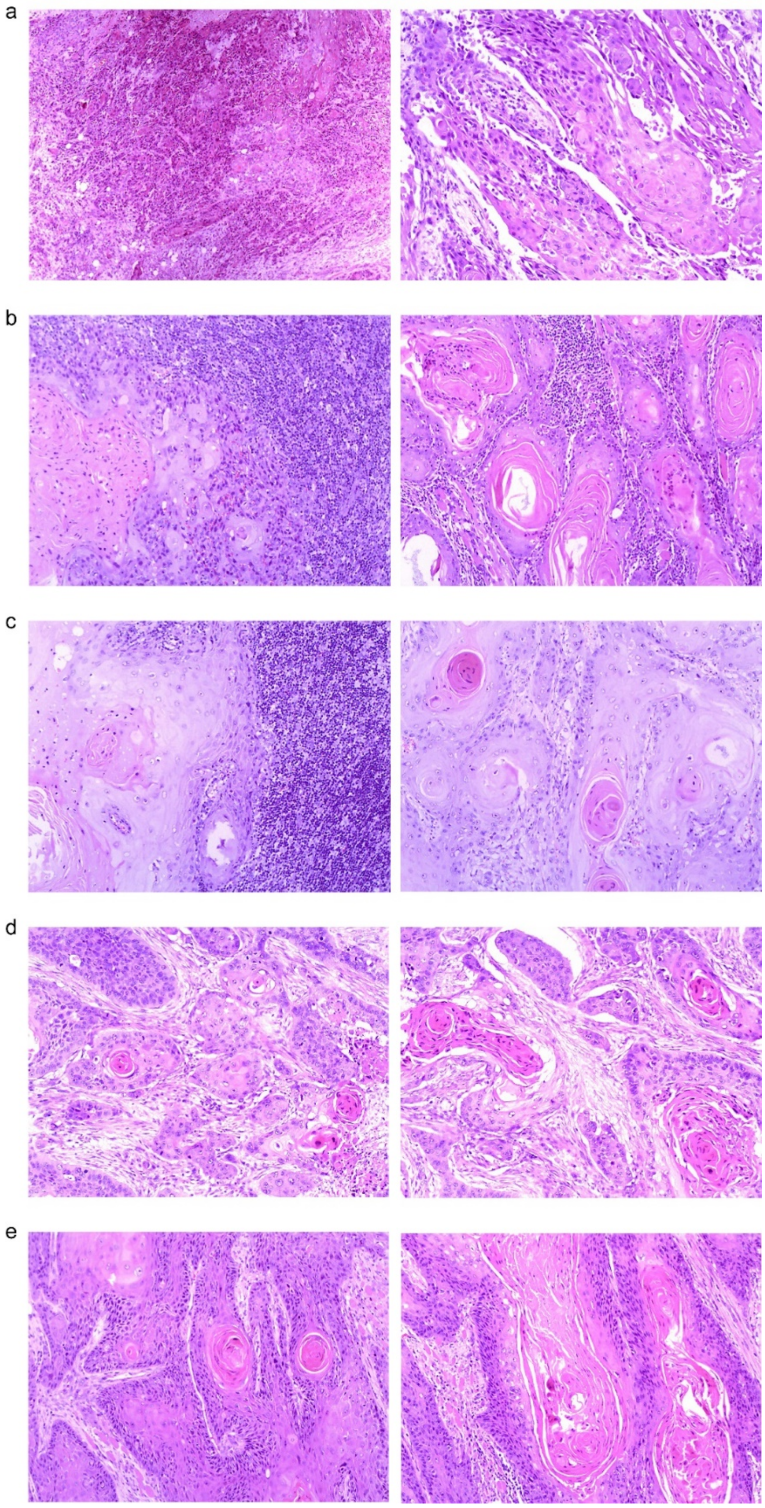

Figure S2. HE staining of cancer tissues from five OTSCC patients. **(a)** 93481, squamous cell carcinoma of the floor of the mouth with squamous carcinoma metastasis detected in bilateral submandibular lymph nodes, and squamous carcinoma metastasis detected in the left deep lower cervical lymph node. **(b)**93541, squamous cell carcinoma of the right tongue, metastasis of squamous carcinoma in the right chin lymph node, and metastasis of squamous carcinoma in the

right deep upper neck lymph node. **(c)** 93510, squamous cell carcinoma of right tongue ventral and tongue root, metastasis of squamous carcinoma in the right deep upper neck lymph node, metastasis of squamous carcinoma in left deep upper neck lymph node. **(d)** 93488, squamous cell carcinoma of the right floor of the mouth with no metastasis in the lymph nodes. **(e)** 93487, squamous cell carcinoma of the right tongue and abdominal floor of the mouth with no metastasis in the lymph nodes.

Table S3 the medical history and clinicopathological characteristics of the 5 patients

Patient-ID	Gender	Age (years)	Smoking history	History of alcohol consumption	Tumor grading	Tumor stage
93481	male	46	25 years, 20 cigarettes/day, quit for 1 month	15 years, 200 g/day, quit for 1 month	T4N2M0	IV
93487	male	48	30 years, 20 cigarettes/day, not quit	25 years, 200 g/day, not quit	T2N0M0	II
93488	male	54	30 years, 10 cigarettes/day, not quit	30 years, 300 g/day, not quit	T3N0M0	III
93510	male	45	20 years, 25 cigarettes/day, quit for 6 months	20 years, 300 g/day, quit for 6 months	T4N2M0	IV
93541	male	29	10 years, 20 cigarettes/day, not quit	No history	T4N2M0	IV

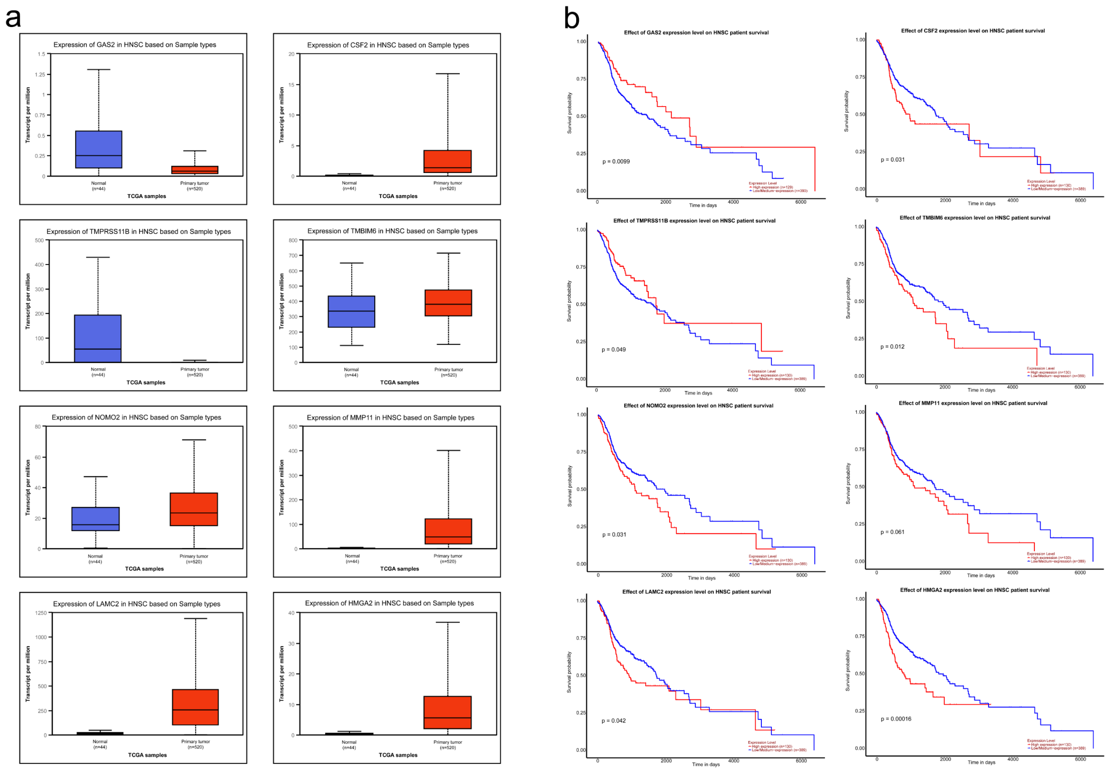

Figure S4. Kaplan–Meier survival curves for the corresponding high- and low-expression cohorts. **(a)** Box plots of gene expression in different tissues. Differential expression of *GAS2*, *CSF2*, *TMPRSS11B*, *TMBIM6*, *NOMO2*, *MMP11*, *LAMC2*, and *HMGA2* across normal and tumor tissues, in that order. **(b)** Kaplan–Meier curves of prognostic survival differences between high- and low-expression groups of *GAS2*, *CSF2*, *TMPRSS11B*, *TMBIM6*, *NOMO2*, *MMP11*, *LAMC2*, and *HMGA2*.

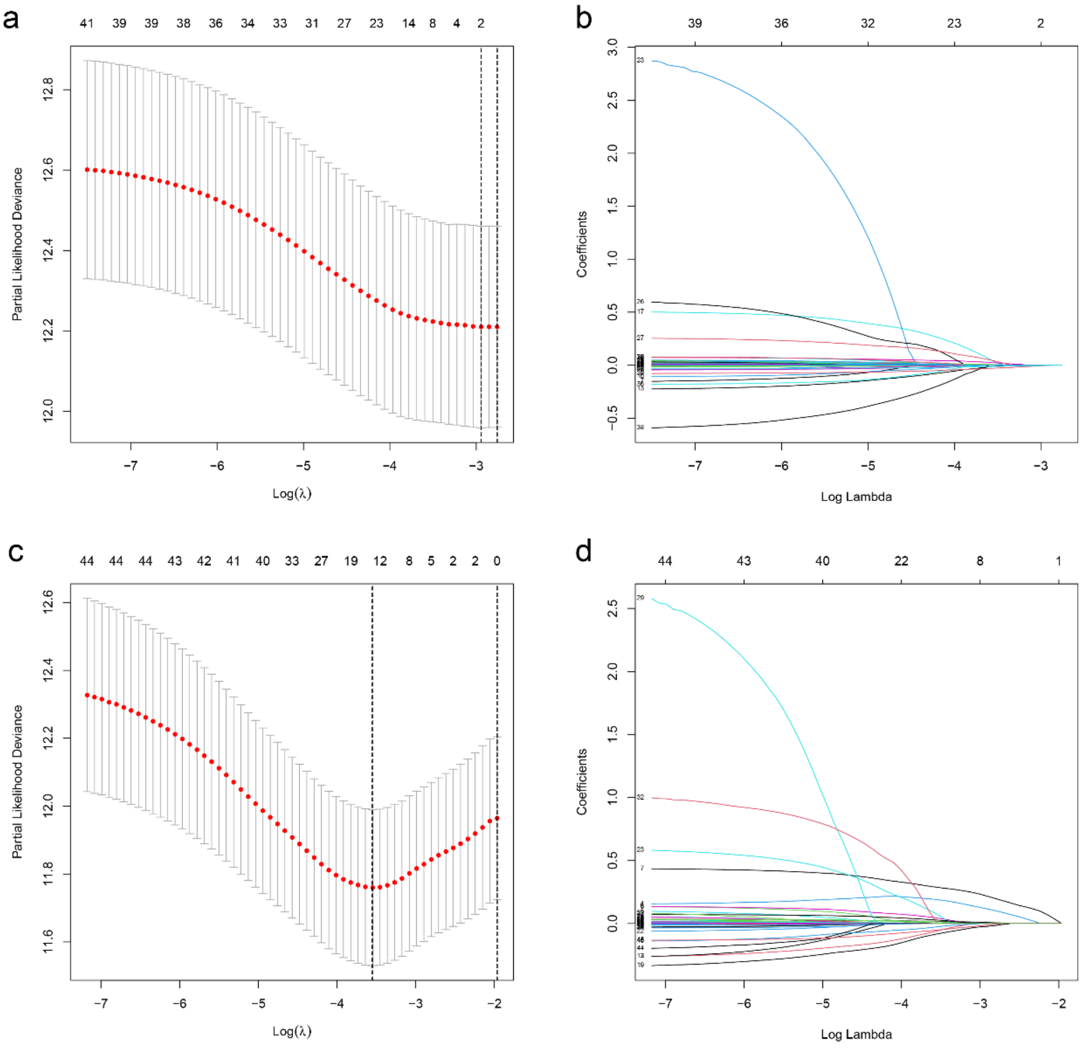

Figure S5. The LASSO regression analysis. **(a)** The first dashed line indicates -3 as the penalty value corresponding to the lowest point of the regression curve. **(b)** Each curve represented a gene. When the penalty value was −3, there were two curves whose slopes were not zero. This graph shows that the results of the LASSO regression analysis contained two genes. **(c)** The lowest point penalty value of the multivariate regression curve is -3.5. **(d)** The results of the LASSO regression analysis contained 14 factors.

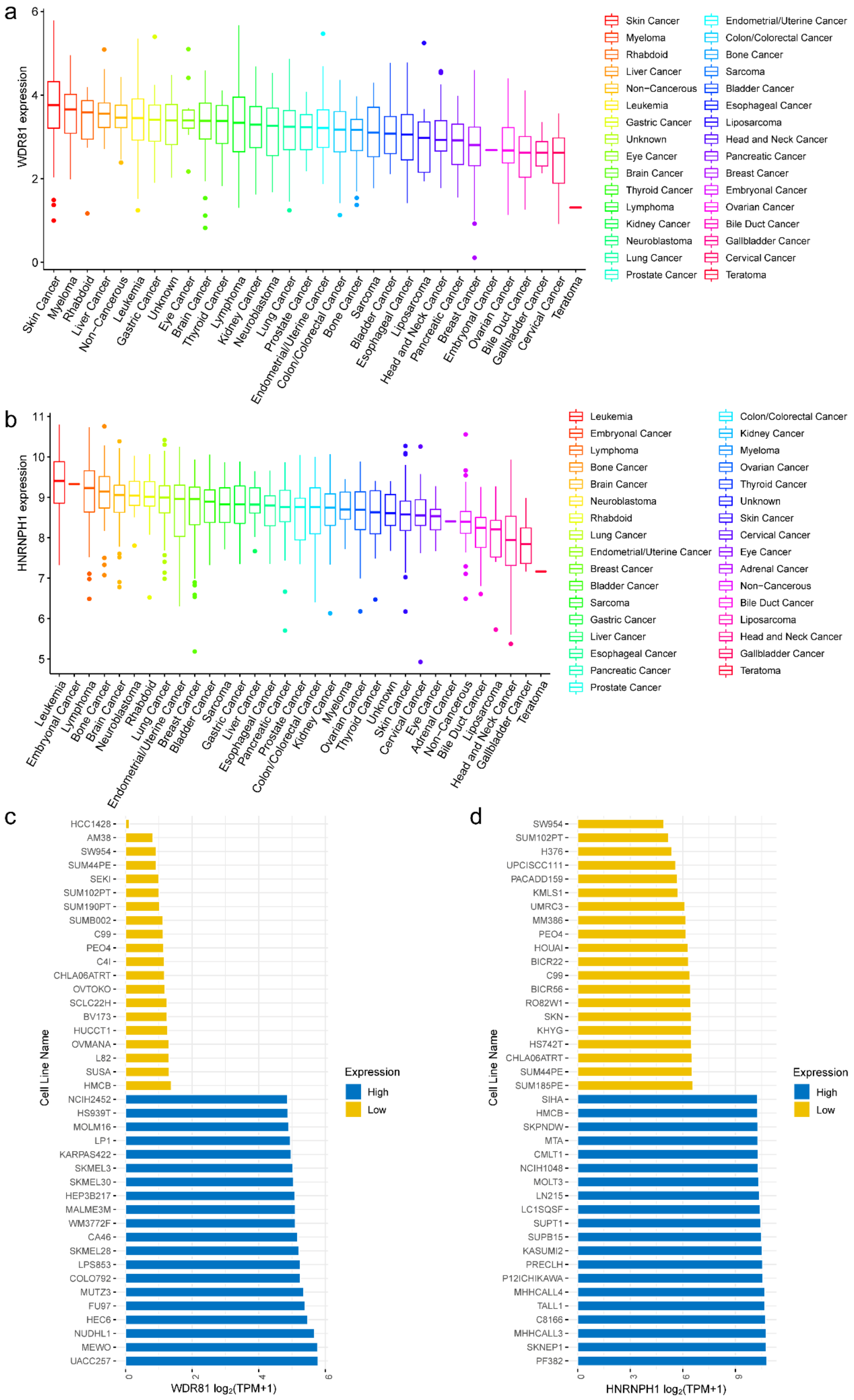
a
WDR81 expression
0
2
4
6
Skin Cancer
Myeloma
Rhabdoid
Liver Cancer
Non−Cancerous
Leukemia
Gastric Cancer
Unknown
Eye Cancer
Brain Cancer
Thyroid Cancer
Lymphoma
Kidney Cancer
Neuroblastoma
Lung Cancer
Prostate Cancer
Endometrial/Uterine Cancer
Colon/Colorectal Cancer
Bone Cancer
Sarcoma
Bladder Cancer
Esophageal Cancer
Liposarcoma
Head and Neck Cancer
Pancreatic Cancer
Breast Cancer
Embryonal Cancer
Ovarian Cancer
Bile Duct Cancer
Gallbladder Cancer
Cervical Cancer
Teratoma
b
HNRNPH1 expression
5
6
7
8
9
10
11
Leukemia
Embryonal Cancer
Lymphoma
Bone Cancer
Brain Cancer
Neuroblastoma
Rhabdoid
Lung Cancer
Endometrial/Uterine Cancer
Breast Cancer
Bladder Cancer
Sarcoma
Gastric Cancer
Liver Cancer
Esophageal Cancer
Pancreatic Cancer
Prostate Cancer
Colon/Colorectal Cancer
Kidney Cancer
Myeloma
Ovarian Cancer
Thyroid Cancer
Unknown
Skin Cancer
Cervical Cancer
Eye Cancer
Adrenal Cancer
Non−Cancerous
Bile Duct Cancer
Liposarcoma
Head and Neck Cancer
Gallbladder Cancer
Teratoma
c
Cell Line Name
HCC1428
AM38
SW954
SUM44PE
SEKI
SUM102PT
SUM190PT
SUMB002
C99
PEO4
C4I
CHLA06ATRT
OVTOKO
SCLC22H
BV173
HUCCT1
OVMANA
L82
SUSA
HMCB
NCIH2452
HS939T
MOLM16
LP1
KARPAS422
SKMEL3
SKMEL30
HEP3B217
MALME3M
WM3772F
CA46
SKMEL28
LPS853
COLO792
MUTZ3
FU97
HEC6
NUDHL1
MEWO
UACC257
0
2
4
6
WDR81 log2(TPM+1)
Expression
High
Low
d
Cell Line Name
SW954
SUM102PT
H376
UPCISCC111
PACADD159
KMLS1
UMRC3
MM386
PEO4
HOUAI
BICR22
C99
BICR56
RO82W1
SKN
KHYG
HS742T
CHLA06ATRT
SUM44PE
SUM185PE
SIHA
HMCB
SKPNDW
MTA
CMLT1
NCIH1048
MOLT3
LN215
LC1SQSF
SUPT1
SUPB15
KASUMI2
PRECLH
P12ICHIKAWA
MHHCALL4
TALL1
C8166
MHHCALL3
SKNEP1
PF382
0
3
6
9
HNRNPH1 log2(TPM+1)
Expression
High
Low

Figure S6. The pan-cancer spectrum and cell lineage of LINC00885 target genes **(a)** Box plot of the WDR81 pan-cancer spectrum. **(b)** Box plot of the HNRNPH1 pan-cancer spectrum. **(c-d)** In cell lineage maps of WDR81 and HNPNPH1, yellow represents the 20 lowest expressing cells, and blue represents the 20 highest expressing cells.

Table S7 the details of the statistical analysis tools

**Software and packages**	**version**	**Open source**
R	4.0.5	
Perl	5.28.1	http://www.perl.org
Cytoscape	3.9.1	https://cytoscape.org/
pheatmap	1.0.12	https://CRAN.R-project.org/package=pheatmap
limma	3.52.3	https://bioconductor.org/packages/limma/
survival	3.3.1	https://CRAN.R-project.org/package=survival
forestplot	2.0.1	https://CRAN.R-project.org/package=forestplot
glmnet	4.1-4	https://CRAN.R-project.org/package=glmnet
survminer	0.4.9	https://CRAN.R-project.org/package=survminer
survivalROC	1.0.3	https://CRAN.R-project.org/package=survivalROC
rms	6.3-0	https://CRAN.R-project.org/package=rms
plyr	1.8.7	https://CRAN.R-project.org/package=plyr
ggpubr	0.4.0	https://CRAN.R-project.org/package=ggpubr
ggplot2	3.3.6	https://CRAN.R-project.org/package=ggplot2
clusterProfiler	4.4.4	https://bioconductor.org/packages/clusterProfiler/
org.Hs.eg.db	3.15.0	https://bioconductor.org/packages/org.Hs.eg.db/
enrichplot	1.12.3	https://bioconductor.org/packages/enrichplot/
GOplot	1.0.2	https://CRAN.R-project.org/package=GOplot
